# Dry Cupping

**Published:** 1875-12

**Authors:** B. H. Washington


### DRY CUPPING.

By B. H. WASHINGTON, M.D.

In the October number of your JOURNAL, I notice a few comments on my article on dry cupping in the May number. The writer says: "It is also true that Dr. Washington in his paper on phlegmasia dolens, has told us that dry cupping on the spinal column would relax the rigid os and external parts, while it would bring on contractions of the fundus; but without some further explanations as to *how* this was probably accomplished, his views might be considered, as a medical friend expressed it to me, while discussing the article, 'too fine spun.'"

I beg leave respectfully to suggest that the proper question for discussion was a question of fact, and not of theory. If my statement that women could have their sufferings shortened by using dry cupping on the spinal column, in such a manner as first to produce relaxation of the os uteri, and contraction of the fundus, was true, it is a matter of no importance whether any mortal on earth ever does understand *how* it can be done.

Facts and explanations are two very different things. The first will bear the severest scrutiny, and the last will not bear any searching investigation, for it is safe to say that not a single one of the phenomena of nature has ever been explained, when thoroughly sifted, the explanation will always be found to be a restatement of facts in other words, and yet we deceive ourselves by fanciful explanations constantly. To say that an apple falls to the ground because of the attraction of gravitation, is no explanation as to *how* or *why* the earth does attract the apple. It is simply that the apple falls downward because it is drawn downwards.

The facts stated can be easily verified by any one not disposed to take Prof. Tenner's corroboration of my statement, and Dr. Hunter's confirmatory statement.

Fact 1. Dry cupping will partially paralyze the muscles of the arm, so as to make them feel heavy if raised to the face.

Fact 2. Dry cupping will partially paralyze the muscles concerned in a dislocation of the humerus, so as to allow of reduction with ease, and almost without pain.

Fact 3. Dry cupping will make the uterus contract. Let any one try it ten or twelve hours after delivery, and he can soon satisfy himself.

Fact 4. Contraction is the natural function of the fundus uteri. No one to dispute.

Fact 5. Expansion, the natural function of the os uteri. No one to dispute.

Fact 6. Dry cupping applied for five to ten minutes on the lower (sacral) portion of the spinal column will produce relaxation of the os uteri and external parts. Any one can test this in a suitable case.

Fact 7. Dry cupping next applied (while the lower cup is still on) above the first cup will produce contraction of the fundus uteri, and that often so as to secure delivery in even less than thirty minutes. Now here are seven facts substantiated by three witnesses (whose testimony would send any man to the penitentiary) on one side, and on the other side an inability to understand *how* these can be explained; and yet hundreds of physicians will refuse to try a remedy because they cannot understand how the fact is to be accomplished. To grapple with the real question of life, not a single soul of such doubters knows any more of "the way of a man with a maid" than old Solomon did, and I must insist that to be logically consistent, such men ought to have separate beds from their wives, until they can clearly explain the origin of life without restating the fact in different words. And yet there is not a single soul of them who will hesitate to get his wife into trouble, though he does not understand how it is accomplished, and then claim to be a wise medical philosopher, because he refuses to get his wife out of trouble, by the use of a remedial agent, whose action he cannot explain. A fig for such wisdom, and such consistency.

I stated that the exposition of natural phenomena are merely restatements of the same facts in different words. Now let us examine a little more closely. My statement of facts was that the natural function of the fundus uteri was to contract, and of the os to expand, and that dry cupping could accomplish both results. Now let us take up the exposition brought up in elucidation of the above dark statements.

The writer says: "Hence as the cervix and vaginal canals are largely supplied from the cerebro-spinal, and the external vaginal and perineal muscles wholly supplied from it, while the fundus of the gravid uterus is almost or entirely under the influence of the sympathetic, we can easily see how anæsthesia would not interfere with the contractions of the uterus, unless long continued, while it would cause relaxation of the cervix and external genitals."

Now those nerves referred to are merely the means by which the functions, to which I referred, are to be performed, and no one can really understand any better than before *how* dry cupping can cause contraction and expansion at the same time, or how anæsthetics can allow both processes to go on at the same time.

To say that opium lulls to sleep is a broad proposition, without reference to any specific part of the system, and when we enquire *why* does opium lull to sleep, instead of the reverse, or *how* it influences the system, we do not know any more than before. When we say that it is done through the medium of its influence on the nervous system, and though we may have every line of Gray and Carpenter on the nervous system committed to memory, still we could not grapple with the real question *how.*

During the first chemical lecture I heard from Professor Proost, he turned litmus paper red by sulphuric acid. After the lecture was over, thinking all wisdom was in his enormous brain, I asked him very politely, "Dr. will you be kind enough to explain to me *how* and why that acid changed the blue to a red?" "Because it is the property of such acid to turn vegetable blues to a red," was his answer. "Yes, that may be so, but it is a fact and not an explanation of a fact. I want to know how and why that acid turned the fluid red?" "Because it is a property of acid to turn vegetable blues to a red," replied he, and he looked at me in wild astonishment at my dullness.

Though knowing that no natural phenomena can really be explained, I have followed the fashion of medical writers, and given a long article to the Confederate States Medical Journal, in which I took Dr. F. Campbell's work on the nervous system, and gave the best explanation I could, secumdum artem; but am sorry to say that the most diligent search has not enabled me to find a file containing the article. In that article I claimed that dry cupping was an excito-secretory remedial agent, as well as an excito-motor, and thereby became a most extraordinary alterative. The cure of a case of tetter of thirty years standing, for which forty doctors had prescribed in vain, by dry cupping alone, and another case of twenty years standing was stated; but after all my exposition, the grand ultimate question *how* was left unexplained. The proximate explanations about the nervous system were made plain enough.

During the war a soldier was discharged from the army for perfect paralysis of the arm from gunshot wound, and this paralysis had existed for months. So far as known to the writer, there is not a single surgical work which would have justified any surgeon in saying he could cure the paralysis. Liniments were useless, and there was not an agent known which would reach the case. Nevertheless the case was cured by dry cupping alone, and the fact that he was paralyzed and cured, can be substantiated by probably one hundred living witnesses here in Augusta.

Suppose, now, I arrange my explanation *secumdum artem*, and affirm that dry cupping excited the absorbent vessels to take up the interstitial deposits, which had kept the ends of the nerves separated, and stimulated the nerves to healthy action, so that new matter was deposited sufficient to fill up the space, and allow nervous communication to be resumed. No one really knows *how* the cure was accomplished any better than he would known upon a simple affirmation that the man was cured.

The important question for consideration is whether it is a fact that dry cupping will influence uterine action, not whether we can understand *how* it influences uterine action, and as it is a question of fact, I refer all parties interested to Professor Bowling, who noticed Dr. Hunter's discovery, and showed that I had anticipated him, and made it known through the pages of his own Journal, and whenever I can get time, I will find, at the medical library, Professor Tenner's report of a case in his own practice, in which he relieved a woman in thirty minutes, who had been suffering thirty hours, and send it to you. I must say I candidly think any man, who will let a woman suffer many hours because he cannot understand *how* she can be relieved, ought to be censurved by all those whose lives are devoted to the relief of suffering humanity.

